# Primary micro neuroendocrine tumor arising in a horseshoe kidney with cyst: report of a case and review of literature

**DOI:** 10.1186/1746-1596-7-126

**Published:** 2012-09-21

**Authors:** Qingfu Zhang, Jian Ming, Siyang Zhang, Xueshan Qiu

**Affiliations:** 1 Department of Pathology, The First Affiliated Hospital and College of Basic Medical Sciences, No. 92 North Second Road, Heping District, Shenyang 110001, China; 2 No. 202 Hospital of People Liberation Army of China, Shenyang, China; 3 Center of Laboratory Technology and Experimental Medicine, China Medical University, Shenyang, China

**Keywords:** Kidney neoplasm, Neuroendocrine tumour, Horseshoe kidney

## Abstract

**Abstract:**

Neuroendocrine tumors are a heterogeneous group of neoplasms that arise from neuroendocrine cells. Primary renal neuroendocrine tumors are among the most unusual of all renal neoplasms, since neuroendocrine cells are not found within normal renal parenchyma. Here, a case of primary micro neuroendocrine tumor (about 4.7 mm*2 mm) arising in the horseshoe kidney with a cyst of a 45-year-old man was reported and a literature review was written.

**Virtual Slides:**

The virtual slide(s) for this article can be found here: http://www.diagnosticpathology.diagnomx.eu/vs/2121156944757267

## Background

Neuroendocrine (NE) tumours include a heterogeneous group of neoplasms arising from NE cells, which are either present in endocrine organs or dispersed through the body, including the gastrointestinal tract, lung, kidney, ovary, testis, et al. The World Health Organization (WHO) classification scheme places neuroendocrine tumors into three main categories: well-differentiated, well-differentiated (low grade) and poorly differentiated (high grade) [1]. The classification of the NE tumours largely depends upon the anatomical site and organ of origin. NE tumours in the lung include four groups of neoplasms with diverse prognosis, i.e. typical and atypical carcinoid, large-cell NE carcinoma (LCNEC) and small cell carcinoma (SCC) [2]. A new classification system for the gastroenteropancreatic NE tumours considers the proliferative activity, with well-differentiated NE tumours and carcinomas being classified as grades 1 and 2, and poorly differentiated NECs are defined as grade 3 [3]. The neuroendocrine (NE) tumours are subdivided into two basic types as carcinoid tumour and neuroendocrine carcinoma in the urinary system and male genital organs. Carcinoid is a rare tumour and similar to its counterpart in other organs, such as lung or gastrointestinal tract. NE carcinoma is extremely rare and also similar to NE carcinoma arising in other organs, which is highly aggressive [4]. Carcinoid tumors in kidney are characteristically low grade malignant tumors with neuroendocrine differentiation. Primary renal neuroendocrine tumors are extremely rare in the world because neuroendocrine cells are not found in normal renal parenchyma, pelvis, and ureter. In the literature, the smallest neuroendocrine tumor is about 2 cm in all cases reported so far.

### Clinical history

A 45-year-old Chinese man was found a microscopic hematuria incidentally by routine urine examination in a medical examination with no other associated symptoms, Computed tomography (CT) scans showed a cystic renal tumor in the left kidney. The patient looked well in appearance and had no specific matter in his medical or family history. There was no evidence of extrarenal invasion or distant metastasis. The horseshoe kidney and renal tumor on the left was revealed by CT. The tumor appeared a cystic mass with high density, about 3.9 cm*4.6 cm in diameter with calcification on plain film, and was obviously enhanced with contrast medium. Ultrasound showed a 4.38 cm*4.59 cm cystic mass in the medial side of the left kidney with unclear boundaries to adjacent organs and no significant color flow was found in the tumor. During the operation, the lower pole of the left kidney was found connected to the right kidney across the abdominal aorta, and the tumor was located close to the renal hilum of the left kidney. The patient underwent a left radical nephrectomy and partly ureter. The size of left kidney is 13 cm*8 cm*6 cm. The length of ureter is 7 cm. The cystic mass measuring 4 cm*4.2 cm*4.5 cm was surrounded by a capsule and the cyst was filled with friable blood clot.

## Materials and methods

### Histologic and immunohistological methods

The surgical specimen was fixed in 10% buffered formalin solution and embedded in paraffin. Histologic sections (4 μm thick) were assessed with hematoxylin and eosin stains. The immunohistochemical staining was performed using the streptavidin-peroxidase system (Ultrasensitive; MaiXin Inc., Fuzhou, China) according to the manufacturer's instruction. Heat-induced epitope retrieval was performed. Commercially available pre-diluted monoclonal antibodies against the following antigens were employed: NSE (1:200; Mouse mAb (4 F12), Merck), Chromogranin A (1:200; Mouse mAb (MAB-0202), MaiXin Inc, Fuzhou, China), Synaptophysin (1:200; Mouse mAb (MAB-0078), MaiXin Inc, Fuzhou, China), P-CK(pan-cytokeratin) (1:200; Mouse mAb (B311.1), Merck), Vimentin (1:200; Mouse mAb (V-9), Merck), CD99 (1:200; Mouse mAb (WLM04), Merck), Desmin (1:200; Mouse mAb (DE-B-5), Merck) and Ki-67 (1:200; MIB1, Dako). The immune reactions were visualized with DAB as the chromogen (Sigma-Aldrich Co., St Louis, Mo, USA). All the internal and external controls worked appropriately.

## Results

### Histologic findings

**Figure 1:**
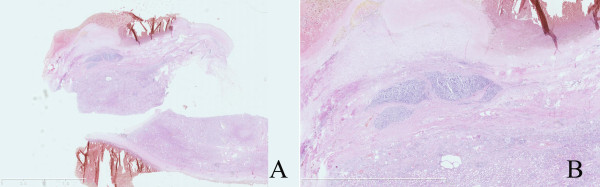
**Cystic spaces component was filled with the blood clots and wasn’t lined by any epithelium; the tumor was located between the cystic spaces and renal cortex,maximum diameter was about 4.7 mm, (Fig A) Original magnification × 3.7.** Scale bar 10 mm. Fig **B** Original magnification × 12.5. Scale bar 5 mm.

**Figure 2:**
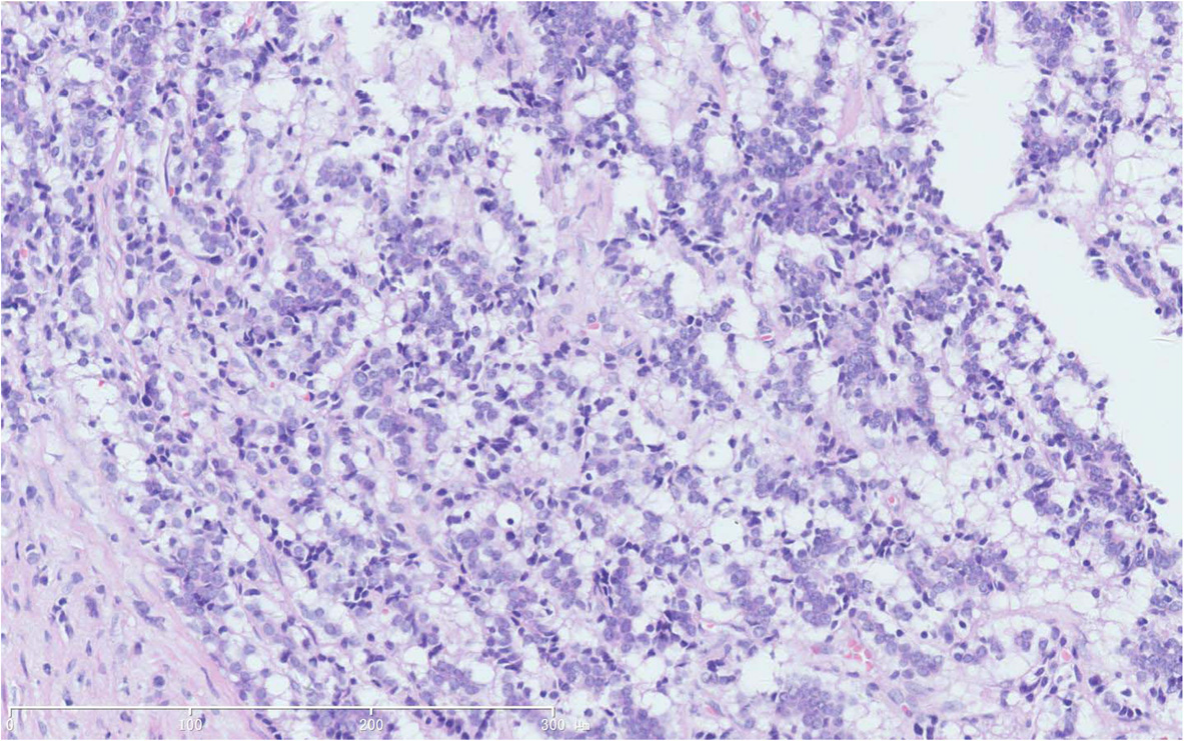
**Tumor showing the classical architectural pattern of trabecular nests of monotonous small round cells with peripheral palisading.** Original magnification × 200.

The histologic examination revealed two components. The first component was cystic spaces filled with the blood clots and was not lined by any epithelium. The tumor was located between the cystic spaces and the renal cortex, about 4.7 mm*2 mm (Figure1). The tumor cells showed the classical architectural pattern of trabecular nests admixed with solid nests within highly vascularized stroma. The tumor cells were largely polygonal with granular clear cytoplasm and indistinguishable cytoplasmic boundaries. The Nuclei were round to oval and uniform in size with rare mitotic figures (Figure2). We dissected the cyst carefully and did not found any more tumors.

**Figure 3:**
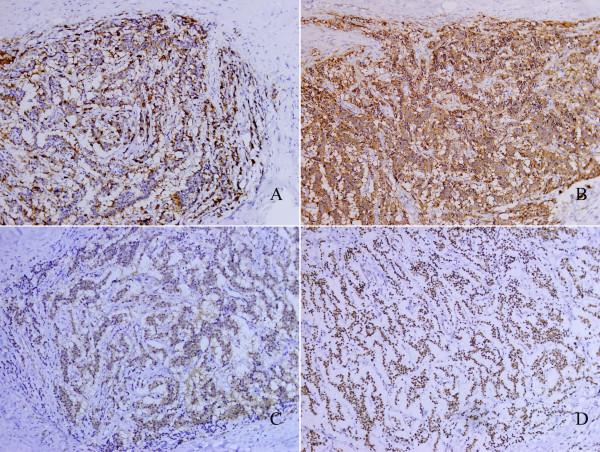
**The tumor cells were positively stained for chromogranin (A); the tumor cells showed cytoplasmic diffuse and strongly expression of synaptophysin (B); the positivity for neuron-specific enolase is weak and focal (C); tumor cells showed necleus diffuse and strongly expression of CD99 (D).** Original magnification × 200.

Immunohistochemical Findings The tumor cells showed cytoplasmic expression of CK (pan) (dispersed). The tumor cells were positively stained for chromogranin (Figure3A). Diffused and strong cytoplasmic immunoreactivity for synaptophysin (Figure3B) were observed. The positivity for NSE was weak and focal (Figure3C). Of interest, CD99 expression was diffused in this case (Figure3D). And the cells were otherwise negative for Desmin and for Vimentin. Less than 1%of the neoplastic cells were positive for Ki-67. No immunoreactive neuroendocrine cell was detected in the nonneoplastic renal parenchyma and renal pelvis/hilum.

## Discussion

Carcinoid tumors are low-grade malignant tumors with neuroendocrine differentiation that are occurring less frequently in the urogenital system. Since the first report of primary renal carcinoid tumor by Resnick in 1966 [5], about 70 cases have been documented in the literature to date. The size of renal carcinoid tumor was 2–30 cm (mean 9.6 cm) in diameter [6,7]. Only 13 cases of primary carcinoid tumor within a horseshoe kidney were found till today in the world. Here, we report a case of 4.7 mm primary micro neuroendocrine tumor (also called carcinoid tumors) arising within the horseshoe kidney. To our knowledge, this is a case of minimum primary renal neuroendocrine tumor presents on the horseshoe kidney.

The primary renal Neuroendocrine tumors have been reported to arise most commonly in the setting of acquired and congenital renal abnormalities (as in the case herein presented), such as horseshoe kidney (18–26 %) [8,9], renal teratoma or teratoid malformation (15 %) [10], and polycystic kidney disease (2 %). Krishnan et al. calculated a relative risk of 62 times for the occurrence of a renal carcinoid tumor in association with a horseshoe kidney [8]. The patient did not found any more tumors in the other organs, which sufficiently confirming the primitivity of this lesion. Accordingly, we concluded that it was another case of renal neuroendocrine tumor of minimum in size found in China.

Neuroendocrine tumors are thought to arise from neuroendocrine cells (NECs) or APUD cells. NECs have been described in the urinary bladder, urethra, and the renal pelvis, but NECs are not found within normal renal parenchyma [11]. So many hypotheses have been proposed for the coexistence of primary renal neuroendocrine tumors, including entrapped neural crest cells in the metanephros during embryogenesis [6], neuroendocrine differentiation of a primitive totipotential stem cell [6,7], and hyperplasia of preexisting neuroendocrine cells within metaplastic or teratomatous epithelium [12-14]. The most popular hypothesis is the totipotent cell hypothesis, that primary renal neuroendocrine tumor arises from totipotential primitive stem cells capable of neuroendocrine, mesenchymal and epithelial differentiation. A recent study showed that primary renal neuroendocrine tumor was absence of the expression of PAX-2 (paired box gene 2) and PAX-8 (paired box gene 8), which are thought to be renal cell lineage specific transcription factors. It may support the theory that these are derived from non-nephrogenic elements [15].

Carcinoid tumorlet has been discribed as a precursor lesion for diffused idiopathic neuroendocrine cell hyperplasia of pulmonary neuroendocrine tumors (typical carcinoid tumors that measurement ≤5 mm), In this case, the tumor was 4.7 mm in maximum diameter, and the patient had no lymph node metastases and systemic endocrine syndrome. Therefore, we considered that this case met the diagnosis of renal carcinoid tumorlet.

The primary treatment for the renal neuroendocrine tumor within a horseshoe kidney is the complete surgical resection, which is curative for localized disease. There was no evidence of recurrence at 8-month follow-up. Further follow-up is needed to evaluate the prognosis of the patient.

## Consent

Consent was received from the patient before publication.

## Competing interests

The authors declare that they have no competing interests.

## Authors’ contributions

QZ drafted the manuscript and performed the literature review, acquired photomicrographs, JM and SZ revised the manuscript for important intellectual content; XQ gave and reviewed the final histopathological diagnosis. All authors read and approved the final manuscript.
